# Mental Health and Psychological Impact during COVID-19 Pandemic: An Online Survey of Portuguese Higher Education Students

**DOI:** 10.3390/ijerph19010337

**Published:** 2021-12-29

**Authors:** Carlos Laranjeira, Maria Anjos Dixe, Olga Valentim, Zaida Charepe, Ana Querido

**Affiliations:** 1 School of Health Sciences, Campus 2, Polytechnic of Leiria, Morro do Lena, Alto do Vieiro, Apartado 4137, 2411-901 Leiria, Portugal; maria.dixe@ipleiria.pt (M.A.D.); olga.valentim@ipleiria.pt (O.V.); 2 Centre for Innovative Care and Health Technology (ciTechCare), Rua de Santo André—66–68, Campus 5, Polytechnic of Leiria, 2410-541 Leiria, Portugal; 3 Research in Education and Community Intervention (RECI I&D), Piaget Institute, 3515-776 Viseu, Portugal; 4 Center for Research in Health and Information Systems (CINTESIS), NursID, University of Porto, 4200-450 Porto, Portugal; 5 Institute of Health Sciences (ICS), Universidade Católica Portuguesa, Palma de Cima, 1649-023 Lisboa, Portugal; zaidacharepe@ucp.pt; 6 Centre for Interdisciplinary Research in Health (CIIS), Universidade Católica Portuguesa, 1649-023 Lisboa, Portugal

**Keywords:** mental health, pandemic, graduation education, Portugal, hope, coping

## Abstract

The COVID-19 pandemic has had significant psychological impact on vulnerable groups, particularly students. The present study aims to investigate the mental and psychological impact of the COVID-19 pandemic and associated factors in a sample of Portuguese higher education students. An online cross-sectional study was conducted among 1522 higher education students selected by convenience sampling. The survey assessed mental health symptoms as well as sociodemographic variables, health-related perceptions, and psychological factors. Results were fitted to binary and multivariable logistic regression models. The overall prevalences of stress, anxiety, and depression were 35.7%, 36.2%, and 28.5%, respectively. Poor mental health outcomes were related with being female, having no children, living with someone with chronic disease, facing hopelessness, and lacking resilient coping. Future studies focusing on better ways to promote mental health and wellbeing among students are warranted. It is necessary to gather more evidence on the post-pandemic mental health using robust study designs and standardized assessment tools.

## 1. Introduction

On 30 January 2020, the World Health Organization (WHO) declared the Coronavirus Disease 2019 (COVID-19) as a public health emergency of international concern, calling on all countries to prepare for containment of the disease [1]. The virus rapidly spread throughout the globe and, on 11 March 2020, the WHO declared the novel coronavirus outbreak as a global pandemic [2]. With no treatment options yet available, several countries adopted healthcare protocols to mitigate the spread of the virus, including measures restricting free circulation and, in some cases, enforcing quarantine and social isolation [3]. In Portugal, a state of emergency was announced on 18 March. Although isolation, quarantine, and social distancing were vital to prevent the spread of the virus, protect peoples’ physical health, and manage medical resources, they may have also led to long-lasting negative consequences, particularly on mental health and wellbeing [4,5,6]. In fact, these preventive measures may have increased the levels of depression and/or anxiety by inducing separation of significant relatives and increasing the perception of isolation and loneliness [7]. 

After a traumatic event, people may suffer from a variety of mental health problems [8], and some pre-existing psychiatric conditions may worsen, such as the risk of suicidality, and neurological and substance use disorders [9,10,11]. The COVID-19 pandemic may be considered a traumatic stressor with crucial negative health implications [12]. Several studies have been performed worldwide to assess the psychological impact of COVID-19 [13,14,15]. Most studies found higher levels of anxiety [16,17,18,19], depression [17,20], stress [21], and complicated grief as a result of the COVID-19 pandemic [22]. The psychological difficulties felt by the population may be due to home confinement, lack of access to psychiatric facilities or inadequate health information [23,24]. Therefore, it is important to provide and reinforce access to social, physical, and emotional support [9]. Research should also focus on gender differences in beliefs and attitudes concerning COVID-19. 

While some research has been done in Portugal, most studies on mental health implications of COVID-19 are related to healthcare workers [25] and the general population [16,26], with few completed studies on higher education students. Evidence on the psychological impact of COVID-19 in this population is particularly important considering this is a critical life-transition period [27] associated with increased rates of anxiety, stress, and depression [28,29]. Moreover, the current pandemic crisis disturbed the life of universities and college campuses, with overwhelming effects on the educational system, social life, and mental health of students [26,30,31].

Risk perceptions and attitudes influence not only decisions but also behaviors, including those directly affecting health, such as exposure to environmental pressures [32]. According to Lazarus and Folkman’s transactional model of stress and coping, individuals are constantly appraising stimuli within their environment and responding emotionally [33]. When stimuli are appraised as stressors, the resulting distress triggers coping strategies to manage emotions or attempt to directly address the stressor itself [32]. 

Resilient coping and hope provide a counterbalance to intrapersonal and interpersonal events that feed anxiety during periods of uncertainty and stress [34,35]. In general, adaptive strategies reduce stress or enhance wellbeing and mental health [36]. The expression of positive emotions is one way resilient individuals adjust psychologically to challenging situation [19,37]. The specific contribution of hope and coping in predicting psychological distress in the context of the COVID-19 pandemic is still unknown.

The mental health of young people has long been recognized as a global public health challenge [38]. Understanding how students react to challenges during a pandemic and how it impacts their psychological adjustment has now assumed central importance. The available evidence indicates elevated psychiatric symptoms among university students in Portugal during quarantine. The study by Maia and Dias [39] explored the levels of anxiety, depression, and stress in Portuguese university students, indicating a significant increase in psychological disturbance during the pandemic period (between suspension of classes and the declaration of a state of emergency in Portugal) compared to normal periods. Another study with students from six higher education institutions in Portugal and Spain, during May 2020, found high perceived stress scores (51.4%) [40]. 

Notwithstanding, there is a lack of knowledge about the impact of mental health problems in higher education students during the COVID-19 pandemic. More studies are needed to estimate prevalence and identify risk and protective factors, in order to improve our understanding of psychological reactions to large-scale infectious disease outbreaks, and ultimately guide policies and intervention strategies to better advise and adjust mental health in vulnerable groups [26,41]. Thus, the main goal of this study is to investigate the mental and psychological impact of the COVID-19 pandemic in a sample of Portuguese higher education students.

The specific aims of this study are as follows: (1) to determine the prevalence and severity of depression, anxiety, and stress; (2) to examine the relation and differences in health-related perceptions, mental health symptoms, and psychological factors, according to the student’s characteristics (gender and level of studies); and (3) to explore factors associated with mental health symptoms (stress, anxiety, and depression).

## 2. Materials and Methods

### 2.1. Study Design

An online cross-sectional design was applied. This study was reported following the Strengthening the Reporting of Observational Studies in Epidemiology (STROBE) checklist. 

### 2.2. Setting and Sample 

Our participants were Portuguese Higher Education students registered in four educational institutions during the academic year 2020/2021. The estimated sample size was obtained from the Raosoft sample size calculator [42]. For a total population of 40,000 students (an average of 10,000 students per institution), with a confidence level of 95%, the sample size should be 381 students. The inclusion criteria were (a) adults (over the age of 18); (b) level of education beyond high school, including undergraduate and graduate programs; (c) living in Portugal at the time of data collection; and (d) able to read and understand Portuguese. Students participating in Erasmus or other mobility programs were excluded.

### 2.3. Data Collection

Using a convenience sampling method, self-reported data were collected between April 2020 to October 2020 through emails and online social networking platforms. This period corresponded to the first wave of COVID-19 in Portugal. The survey was delivered via Google Forms. Thus, the online distribution of the survey enabled fast access by a large number of students. A network IP address restriction was set to prevent someone from answering multiple times. A pilot study, with a group of 20 students, validated the content and anonymization, and gathered user feedback. Completion of the survey lasted about 10 min. After addressing minor bugs and reformulating questions to avoid possible common method biases caused by wording and distribution of items, the survey was deployed more widely. To increase responses rates, the study was designed to send follow-up reminders every 30 days during the study period. During survey activation, we obtained 390 responses, of which 29 were deemed incomplete and excluded. The invitation to participate was also emailed to 2000 potential participants using institutional mailing lists. After checking, only 1161 responses were considered valid. Thus, overall, 1522 participants completed the survey (a response rate of 64%).

### 2.4. Instruments

The e-questionnaire was divided into five sections. The first section covered the sociodemographic and academic characteristics of the students, including age, gender, marital status, children, living with someone with a chronic condition, present level of study, if they were working students, type of educational institution, study area, and if educational activity continued during the pandemic. 

The second section asked students about their perception of getting COVID-19, and perception of mental, physical, and global health. Participants answered on a scale from 1 (lowest value) to 10 (highest value), using the last month as a reference.

This was followed by a section on how frequently they felt agitated, anxious, down, or sad due to physical distance measures (staying at home, closing schools and universities, closing non-essential goods, etc.). Participants answered on a four-point Likert scale ranging from 1 (never) to 4 (every day). 

The fourth section concerned participant mental health status, using the Portuguese version of the Depression, Anxiety, and Stress Scale (DASS-21) [43,44]. The total depression subscale score was divided into normal (0–9), mild (10–12), moderate (13–20), severe (21–27), and extremely severe depression (28–42). The total anxiety subscale score was divided into five categories [45]: normal (0–6), mild (7–9), moderate (10–14), severe (15–19), and extremely severe anxiety (20–42). Finally, the total stress subscale score was divided into normal (0–10), mild (11–18), moderate (19–26), severe (27–34), and extremely severe stress (35–42). This questionnaire is a reliable and valid measure for assessing the mental health of a Portuguese-speaking population [43]. In our study, the Cronbach’s alpha coefficient of the DASS-21 was 0.923 for the stress scale, 0.891 for the anxiety scale, and 0.922 for the depression scale. 

The last section concerned psychological factors, including hope and resilient coping. The hope levels were assessed through the Portuguese version of the Herth Hope Index (HHI) [46,47]. The HHI measures different dimensions of hope, using a four-point Likert rating scale ranging from 1 (strongly disagree) to 4 (strongly agree). The scale has an overall score ranging from 12 to 48, where a higher score indicates a higher level of hope. Cronbach’s alpha for the present study was 0.875. Resilience was defined as an individual’s ability to recover from stress despite significant adversity. Participants’ tendency to cope adaptively was assessed using the Portuguese version of the Brief Resilient Coping Scale [BRCS] [48,49]. This is a four-point measure, with the following cut-off points: low resilience from 4 to 13 points; medium resilience from 14 to 16 points; high resilience from 17 to 20 points. The instrument showed high-reliability rates, with a Cronbach’s alpha coefficient of 0.815.

### 2.5. Ethical Considerations

The protocol of the study was formally approved by the local ethics committee (CE/IPLEIRIA/22/2020). As research data involved human participants, the study was conducted according to the Helsinki Declaration guidelines [50]. Personal data was processed following the EU General Data Protection Regulation (GDPR2016/679). Only the research manager had access to data identifying the participants. The full questionnaire began with information indicating: (1) the purpose of the study; (2) that participation is voluntary; (3) that participation can be terminated at any time, and (4) that all data is handled confidentially. Consent from each student was obtained at the beginning of each online survey. No monetary gifts were given for completing the questionnaire. 

Administration of the survey considered methods to reduce bias due to social desirability, acquiescence, leniency effects; guaranteeing anonymity; and encouraging respondents to answer honestly. 

### 2.6. Data Analysis

Descriptive data analysis was performed using frequencies, percentages, mean ± standard deviation (SD), and ranges. Pairs of groups were compared using independent samples *t*-tests for continuous variables. One-way analysis of variance (ANOVA) was performed to evaluate the significance of differences between more than 2 groups. When the effect of ANOVA was significant, the Games-Howell post-hoc tests were used to detect which groups were different. 

The association between mental health outcomes with each covariate was assessed with bivariate logistic regression to identify potential candidates for multivariate logistic regression [51]. Those variables having a *p*-value less than 0.05 were entered into the multivariate logistic regression model. Cut-off points were found for each outcome variable: ‘depressed’ individuals with scores greater than nine; ‘anxious’ individuals with scores greater than seven; and ‘stressed’ individuals with scores greater than 14. When a significant independent variable was not a continuous variable, it was converted to a dummy variable for use. Odds ratios (OR) with the corresponding 95% confidence interval (95%CI) were computed to assess the strength of association [51]. Data were analyzed using Statistical Package for the Social Science (SPSS version 27 for Windows, IBM Corp., Chicago, IL, USA). 

## 3. Results

### 3.1. Sociodemographic and Academic Characteristics of Participants 

A total of 1522 students (75.1% women and 24.9% men) took part in this investigation. Full demographic and academic characteristics are presented in Table 1. The ages ranged from 18 to 59, with a mean age of 22.88 ± 6.93 years. Most participants were single (91.2%), had no children (93%), and did not live with someone with a chronic condition (73.7%). Regarding the level of studies, most students attended the Public Polytechnic Higher Education (73.7% at the undergraduate level) and were not working while studying (76.6%). In terms of field of study, about 95% of students were enrolled in programs other than health science. Due to the pandemic, most of the students maintained distance teaching, learning, and training activities (80.9%).

### 3.2. Mental Health Status, Psychological Factors, and Health-Related Percetions

Participants reported a moderate risk (51.9%) of getting COVID-19 infection (Table 2). Regarding their perception of mental, physical, and global health during the last month, students reported a range of 6.16 ± 2.14 regarding mental health, 6.51 ± 1.99 for physical health, and 6.69 ± 1.79 for global health. Around 55% of the students spent a few days feeling agitated, anxious, down, or sad due to physical distance measures. Among the participants, 35.7%, 36.2%, and 28.5% had symptoms of stress, anxiety, and depression above the normal range, respectively. High resilience scores were found in 215 participants (14.1%). The mean hope (HHI) was 35.53 (SD = 5.92). 

### 3.3. Comparisons between Study Variables According to the Gender and Level of Studies

Comparisons between genders and levels of study are displayed in Table 3. There were gender differences in mental health perception, with females presenting lower levels than males [t(1522) = 2.49, *p* = 0.013]; in physical health perception, with females presenting lower levels than males [t(1522) = 1.98, *p* = 0.048]; and global health perception, with females presenting lower levels than males [t(1522) = 2.29, *p* = 0.022]. There were also gender differences in stress [t(1522) = −5.71, *p* < 0.001] and anxiety [t(1522) = −4.77, *p* < 0.001], with females presenting higher levels than males in both variables. There were no gender differences in depression [t(1522) = −1.65, *p* = 0.099]. Gender differences were also found in hope levels, with females presenting higher levels than males [t(1522) = −2.19, *p* = 0.029]. No gender differences were found in resilient coping [t(1522) = 1.45, *p* = 0.146]. 

There were differences among participants according to their present level of study. Undergraduate students presented higher levels of stress than students attending other levels of study [F(1, 1521) = 3.52, *p* = 0.015]. Undergraduate students presented lower levels of hope compared to Master’s students, who presented higher levels of hope when compared to other levels of studies [F(1, 1521) = 4.73, *p* = 0.009]. Undergraduate students presented lower levels of resilience when compared to Master’s students [F(1, 1521) = 6.75, *p* < 0.001]. Moreover, students in other levels of study presented lower levels of resilience when compared to postgraduate and master’s students.

### 3.4. Predictors of Mental Health Status

A binary regression analysis assessed the association between dependent and independent variables, as displayed in Table 4. Female scores were significantly higher than males on measures of stress and anxiety, but not depression. Students with no children had a significantly lower risk of stress, anxiety, and depression. Students studying health sciences had a higher risk of depression. Participants who did not work had a significantly lower risk of stress, anxiety, and depression.

Participants who were living with someone with a chronic condition had a significantly higher risk of stress and anxiety, but not depression. Additionally, participants lacking hope or resilient coping had a significantly higher risk of stress, anxiety, and depression.

The multivariable logistic regression revealed that the odds of stress were strongest among female college students (OR = 1.930; 95%CI: 2.533–1.470), with no children (OR = 0.349; 95%CI: 0.607–0.201), living with someone with chronic disease (OR = 1.318; 95%CI: 1.685–1.031), and feeling hopelessness (OR = 3.767; 95%CI: 4.974–2.853). 

The odds of feeling anxiety were strongest among female college students (OR = 1.848; 95%CI: 2.435–1.403), with no children (OR = 0.279; 95%CI: 0.511–0.152), living with someone with chronic illness (OR = 1.289; 95%CI; 1.655–1.003), feeling hopelessness (OR = 4.751; 95%CI: 6.390–3.532), and lacking resilient coping (OR = 1.372; 95%CI: 1.752–1.074).

Finally, high levels of depression were positively associated with hopelessness (OR= 12.425; 95%CI: 17.166–8.994); lack of resilient coping (OR = 1.523; 95%CI: 2.035–1.140); not having children (OR = 0.241; 95%CI: 0.526–0.110) and studying health sciences (OR = 1.436; 95%CI: 1.897–1.087).

## 4. Discussion

The present study detected levels of depression, anxiety, and stress among higher education students that are in line with other studies [8]: 17.2%, 23.6%, and 6% of participants reported moderate to extremely severe symptoms of depression, anxiety, and stress, respectively. However, these results are somewhat lower than those reported in previous COVID-19 Portuguese studies [36,52], especially for depression and stress. In contrast, the severity of mental health symptoms was higher than in Paulino et al. [26]. Similar with Ferreira et al. [36] these differences may be related to when data was collected. While the current study collected the data between April and October 2020, the previous studies had collected their data in late March—after the beginning of the first national lockdown. 

Overall, most of our sample reported normal levels of depression, anxiety, and stress. These results contradict several published studies performed during the COVID-19 pandemic that assessed its psychological impact and addressed its negative psychological consequences [13,14,16,20,53,54]. Differences may be due to the instruments used to measure variables, the sample size, the curricular load, and the existing socio-cultural differences among countries [55,56]. Considering these contradictory findings, it is important to continue to study this population (university students), namely trying to better understand the possible resilience factors that may buffer the effects of the COVID-19 pandemic. 

Nevertheless, the researchers suggest that, in some people, the isolation, fear of contracting SARS-CoV-2, and uncertainty during the pandemic may have led to the collection of symptoms that make up health anxiety linked to COVID. This condition is a relatively new concept, manifests as the inability to leave the house because of COVID-19 fears, frequent checking for symptoms despite not being in a high-risk scenario, and avoiding social situations or people [57]. This issue needs further investigation.

Participants reported a relatively good average perception of mental, physical, and global health. This result reveals that most participants did not negatively report their mental health. Moreover, most students spent only a few days feeling agitated, anxious, down, or sad due to physical distance measures, suggesting they may have found mechanisms to overcome the negative consequences of physical distance measures. This result is relevant, as greater self-perceived health has been associated with a greater experience of depression, anxiety, and psychological distress in various populations [58]. This reinforces the importance of research on positive outcomes and protective factors [59]. 

Several group differences were found. For instance, females presented lower levels of perception of mental, physical, and global health than men, in line with previous research in the Portuguese population during the COVID-19 pandemic [19,53,54]. Moreover, this tendency is typical in the literature from before the COVID-19 pandemic [60], suggesting these differences remained even during a pandemic situation. Several studies conducted during the pandemic have confirmed the greater psychological vulnerability in women compared to men [58,61,62]. Although females reported reduced perception of mental, physical, and global health, it is noteworthy that females presented higher levels of hope than men. This may seem contradictory, but can be explained given the nature of hope, as described in the literature: describing oneself as being in a hopeless state with poorer self-perceived health, does not mean that hope has entirely vanished [63]. 

Some interesting results were also found when comparing participants regarding their attending level of study. Undergraduate students presented higher levels of stress than other students. Differences were also found in levels of hope and resilient coping, with undergraduate students presenting lower levels compared to master’s students, and master’s students presenting higher levels of hope and coping. This is consistent with previous studies reporting that students expressed a need for emotional and psychological support mainly to maintain high performance levels, which can be explained by resilience theory [64]. Academically engaged students tend to be more internally resilient [65] and capable of adapting to changes brought about by external threats and perceived crisis, better known as the need–threat internal resilience [66]. Resilient coping is strongly related with perceived emotional support and close, safe relationships [67]. These networks of emotional and social support serve as buffers to stress and its negative consequences, and provide resources for coping with stress, thus helping to curtail more severe problems.

Our results provide useful insights into the predictors of mental health during the COVID-19 pandemic. Poor mental health outcomes seems to be related with being female, having no children, living with someone with chronic disease, studying health sciences, feeling hopelessness and lacking resilient coping. Some associations were reported in previous studies involving similar samples [68]. Women carry a heavier burden to ensure a family’s overall wellness, and feel greater pressure with working and/or studying, as this requires a balance between work and family commitments [69]. Therefore, having children and living with relatives with a chronic disease may increase the fear and anxiety related with COVID-19 [65], in addition to the societal responsibility already imposed on a woman [69].

Typically, students from health sciences experience considerable stress and depressive symptoms during their training, precipitated by many factors, such as adjustment to the novel school environment, information overload, lack of leisure time, financial constraints, family-related stressors, and intense competition for higher grades [70]. Restrictions due to the pandemic delayed numerous clinical teachings and consequently the development/completion of courses, further aggravating the students’ signs of distress. The rapid changes imposed upon higher education students, from the suspension of classes to the declaration of a state of emergency, triggered difficulties in adaptation and less positive emotional states [40]. 

Another interesting finding was that hope and resilient coping had protective effects by reducing the probability of experiencing depression, anxiety, and stress. One recent study [71] showed that hope can lead to positive outcomes, even during a pandemic, as having more positive expectations regarding the future and feeling one leads a meaningful life can mediate how basic health predicts levels of stress, such as those generated by the pandemic. Students have demonstrated the incredible human capacity to reorient under new conditions. Hope seems to be closely linked to wellbeing, as hopeful people approach stressful events in healthier ways. In addition to this innate potential, flexibility and creativity should be emphasized in educational training and institutions can steer education models toward more adaptable practices [72]. 

The current study has significant implications for practice and policy. First, developing programs to improve mental health should be a priority in a government’s response to this pandemic crisis [36]. These plans need to be cross-sectoral, combining measures to protect and promote mental health with actions to treat additional mental ill-health [73]. Second, communication is an important resource in dealing with mental health issues. Higher education institutions play an important role by providing students with educational materials, but also with opportunities to interact with peers and teachers [74]. Universities should also provide guidelines and principles for effective online learning and should ensure that content meets educational requirements, while not overburdening students. Third, healthcare services can communicate with students through social media platforms to help them cope with mental health issues [74]. This can help to maximize the opportunities to reach all individuals in mental distress through psychological counseling services [73]. Restrictions required that psychological and psychiatric support adopt strategies such as remote tools, including videoconferences or other web-based technologies [52,75]. Finally, as hope can help deal with uncertainties, difficulties, and stressful situations, it is important to research hope and that institutions and societies adopt a language of hope during situations of crisis, and even develop specific hope-focused interventions to mitigate the psychological impact of this and future pandemics [63].

### Limitations of the Study

The present study has several limitations that should be noted. First, the cross-sectional design does not provide solid and causal evidence for detected associations. The variables studied can be better understood with longitudinal designs. Second, our final sample comprised mainly female undergraduate students recruited online. A previous study indicated that social media particularly attracts people who are distressed, looking for support [76]. This could bias the data and hinder any representativeness of our sample. This fact, as well as a non-probability sampling technique, decreased the external validity of our study. Additionally, data collection covered a large temporal window (7 months). When our data collection ended (October 2020), fear and anxiety related to COVID-19 was less present in the public consciousness than at the beginning of the pandemic, and thus less likely to have been a major factor influencing the presentation of mental health symptoms.

Due to the anonymous nature of responses, the study could not assess whether non-responders might be students with little interest in the issue of “mental health in the time of COVID-19”. The nonresponse bias cannot be investigated online, since the identity of non-respondents is generally unknown [77]. Future studies should integrate a subsequent follow-up system in their procedure, as proposed in Rogelberg and Stanton [78], to assess possible non-response bias. 

Despite these limitations, the current study has a number of strengths such as (a) the potential contribution to the mental health field; (b) the use of standard and validated self-report measures that allow the assessment of study variables; and (c) a good sample size. Finally, the study does not focus solely on negative variables, but assesses also positive variables, such as hope and resilient coping. 

## 5. Conclusions

This study found a moderate prevalence of stress, anxiety, and depression in higher education students and highlighted some risk factors significantly associated with these symptoms. These risk factors should be addressed by the respective stakeholders (e.g., university teachers, healthcare practitioners, and public health policymakers) to facilitate a compassionate and mental health-friendly environment for higher education students.

## Figures and Tables

**Table 1:** Sociodemographic and academic characteristics (*n* = 1522).

Variables	Categories	*n* (%)
**Age group**	18 to 24 years old	1233 (81.0)
	25 to 65 years old	289 (19.0)
**Gender**	Male	379 (24.9)
	Female	1143 (75.1)
**Marital status**	Single	1388 (91.2)
	Married	118 (7.7)
	Divorced	13 (0.9)
	Widower	3 (0.2)
**Having children**	No	1416 (93.0)
	Yes	106 (7.0)
**Living with someone with a chronic condition**	No	1121 (73.7)
	Yes	401 (26.3)
**Present level of study**	Undergraduate	1205 (79.2)
	Postgraduate studies	15 (1.0)
	Master’s degree	154 (10.1)
	Other	148 (9.7)
**Working student**	No	1166 (76.6)
	Yes	356 (23.4)
**Type of educational institution**	Public University	162 (10.6)
	Public Polytechnic	1273 (83.5)
	Private University	59 (3.9)
	Private Polytechnic	28 (1.8)
**Study areas**	Health Sciences	545 (35.8)
	Not Health Sciences	977 (64.2)
**Students maintained educational activity**	No	291 (19.1)
	Yes	1231 (80.9)

**Table 2:** Mental health status, psychological factors, and health-related perceptions of participants (*n* = 1522).

Variables	Categories	*n* (%)
**Perception of risk of getting COVID-19**	No risk	13 (0.9)
	Low risk	332 (21.8)
	Moderate risk	790 (51.9)
	High risk	250 (16.4)
	Don’t know	137 (9.0)
**Frequency of psycho-emotional symptoms**	Never	141 (9.3)
	A few days	839 (55.1)
	Almost everyday	402 (26.4)
	Everyday	140 (9.2)
**Resilient coping (BRCS)**	Low resilience (4–13)	971 (63.8)
	Medium resilience (14–16)	336 (22.1)
	High resilience (17–20)	215 (14.1)
**DASS-Depression**	Normal depression (0–9)	1088 (71.5)
	Mild depression (10–12)	173 (11.4)
	Moderate depression (13–20)	246 (16.2)
	Severe depression (21–27)	15 (1.0)
	Extremely severe depression (28–42)	0 (0)
**DASS-Anxiety**	Normal anxiety (0–6)	971 (63.8)
	Mild anxiety (7–9)	191 (12.5)
	Moderate anxiety (10–14)	234 (15.4)
	Severe anxiety (15–19)	101 (6.6)
	Extremely severe anxiety (20–42)	25 (1.6)
**DASS-Stress**	Normal stress (0–10)	978 (64.3)
	Mild stress (11–18)	452 (29.7)
	Moderate stress (19–26)	92 (6.0)
	Severe stress (27–34)	0 (0)
	Extremely severe stress (35–42)	0 (0)
**Variables**		**mean ± SD (min–max)**
**Hope (HHI)**		35.53 ± 5.92 (12–48)
**Perception of mental health during last month**		6.16 ± 2.14 (1–10)
**Perception of physical health during last month**		6.51 ± 1.99 (1–10)
**Perception of global health during last month**		6.69 ± 1.79 (1–10)

**Table 3:** Comparisons between health-related perceptions, mental health symptoms, and psychological factors, according to the student’s characteristics (gender and level of study).

	Gender			Present Level of Study							
**Variables**	**Male** ***n* = 379** **M (SD)**	**Female** ***n* = 1143** **M (SD)**	**Total** ***n* = 1522** **M (SD)**	**t**	**Undergraduate** ***n* = 1205** **M (SD)**	**Postgraduate Studies** ***n* = 15** **M (SD)**	**Master’s Degree** ***n* = 154** **M (SD)**	**Other** ***n* = 148** **M (SD)**	**Total** ***n* = 1522** **M (SD)**	***F***	**Post-Hoc**
Perception of mental health	6.39 (2.24)	6.08 (2.10)	6.16 (2.14)	2.49 *	6.11 (2.14)	6.80 (2.34)	6.23 (2.06)	6.42 (2.15)	6.16 (2.14)	1.46	-
Perception of physical health	6.68 (2.07)	6.45 (1.96)	6.51 (1.99)	1.98 *	6.48 (1.99)	7.20 (1.78)	6.52 (1.94)	6.70 (2.08)	6.51 (1.99)	1.15	-
Perception of global health	6.87 (1.82)	6.63 (1.78)	6.69 (1.79)	2.29 *	6.65 (1.78)	7.20 (1.66)	6.64 (1.84)	6.95 (1.86)	6.69 (1.79)	1.68	-
Stress	6.98 (5.64)	8.91 (5.72)	8.43 (5.76)	−5.71 ***	8.64 (5.71)	7.33 (5.49)	8.13 (5.93)	7.10 (5.84)	8.43 (5.76)	3.52 *	1 > 4
Anxiety	4.56 (4.84)	6.06 (5.46)	5.69 (5.35)	−4.77 ***	5.89 (5.39)	4.53 (4.78)	4.88 (5.15)	5.01 (5.12)	5.69 (5.35)	2.75 *	-
Depression	6.16 (5.64)	6. 71 (5.60)	6.57 (5.61)	−1.65	6.76 (5.62)	5.53 (5.48)	5.99 (5.50)	5.78 (5.59)	6.57 (5.61)	2.13	-
Hope	34.95 (6.32)	35.72 (5.78)	35.53 (5.92)	−2.19 *	35.41 (5.86)	37.93 (6.60)	36.95 (5.77)	34.80 (6.27)	35.53 (5.92)	4.73 **	1 < 3 3 > 4
Resilient coping	12.73 (3.51)	12.43 (3.46)	12.51 (3.48)	1.45	12.45 (3.38)	15.00 (3.59)	13.29 (3.63)	11.93 (3.88)	12.51 (3.48)	6.75 ***	1 < 3 2 > 4 3 > 4

* *p* < 0.05; ** *p* < 0.01; *** *p* < 0.001.

**Table 4:** Regression analysis of variables by stress, anxiety, and depression among students (*n* = 1522).

Variables		Stress (DASS-S > 14)			Anxiety (DASS-A > 7)			Depression (DASS-D > 9)		
		Yes/No	OR (95%CI) †	*p*-Value	Yes/No	OR (95%CI) †	*p*-Value	Yes/No	OR (95%CI) †	*p*-Value
**Gender**										
Male		101/278	1 [Reference]	*p* < 0.001	107/272	1 [Reference]	*p* < 0.001	108/271	1 [Reference]	*p* > 0.05
Female		443/700	1.742 (1.347–2.252)		444/699	1.615 (1.253–2.080)]		326/817	1.001 (0.774–1.295)	
**Having children**										
Yes		16/90	1 [Reference]	*p* < 0.001	13/93	1 [Reference]	*p* < 0.001	8/98	1 [Reference]	*p* < 0.001
No		528/888	0.299 (0.174–0.514)		538/878	0.228 (0.126–0.412)		426/990	0.190 (0.091–0.393)	
**Study area**										
Health Sciences		183/362	1.159 (0.930–1.445)	*p* > 0.05	181/364	1.226 (0.984–1.528)	*p* > 0.05	121/424	1.652 (1.296–2.105)	*p* < 0.001
Not Health Sciences		361/616	1 [Reference]		370/607	1 [Reference]		313/664	1 [Reference]	
**Working student**										
Yes		103/253	1 [Reference]	*p* < 0.01	103/253	1 [Reference]	*p* < 0.001	77/279	1 [Reference]	*p* < 0.001
No		441/725	0.669 (0.517–0.866)		448/718	0.652 (0.504–0.844)		357/809	0.625 (0.472–0.829)	
**Living with someone with a chronic condition**										
Yes		165/236	1.369 (1.083–1.730)	*p* < 0.01	165/236	1.331 (1.054–1.682)	*p* < 0.05	128/273	1.249 (0.975–1.600)	*p* > 0.05
No		379/742	1 [Reference]		386/735	1 [Reference]		306/815	1 [Reference]	
**Maintenance of educational activity**										
Yes		452/779	1.255 (0.955–1.649)	*p* > 0.05	452/779	1.125 (0.860–1.472)	*p* > 0.05	128/273	1.065 (0.800–1.416)	*p* > 0.05
No		92/199	1 [Reference]		99/192	1 [Reference]		306/815	1 [Reference]	
Resilient coping *										
Yes (0)		155/399	1 [Reference]	*p* < 0.001	147/404	1 [Reference]	*p* < 0.001	92/459	1 [Reference]	*p* < 0.001
No (1)		389/582	1.708 (1.363–2.140)		404/567	1.958 (1.559–2.459)		342/629	2.713 (2.092–3.517)	
Hope *										
Yes (0)		870/375	3.630 (2.771–4.756)	*p* < 0.001	881/364	5.029 (3.803–6.651)	*p* < 0.001	1022/223	14.652 (10.724–20.018)	*p* < 0.001
No (1)		108/169	1 [Reference]		90/187	1 [Reference]		68/211	1 [Reference]	

† Adjusted OR (odds ratio); CI (confidence interval); * Dummy variable (0 = mental health protector; 1 = mental health non-protector).

## Data Availability

All data generated or analyzed during this study are included in this article.

## References

[1] World Health Organization (2020). Novel Coronavirus (2019-nCoV): Situation Report-10.

[2] World Health Organization (2020). Novel Coronavirus (2019-nCoV): Situation Report-51.

[3] World Health Organization (2020). Critical Preparedness, Readiness and Response Actions for COVID-19—Interim Guidance.

[4] Brooks S., Webster R., Smith L., Woodland L., Wessely S., Greenberg N., Rubin G. (2020). The psychological impact of quarantine and how to reduce it: Rapid review of the evidence. Lancet.

[5] Ochnik D., Rogowska A., Kuśnierz C., Jakubiak M., Schütz A., Held M., Arzenšek A. (2021). Mental health prevalence and predictors among university students in nine countries during the COVID-19 pandemic: A cross-national study. Sci. Rep..

[6] Rahman M.A., Islam S.M.S., Tungpunkom P. (2021). COVID-19: Factors associated with psychological distress, fear, and coping strategies among community members across 17 countries. Glob. Health.

[7] Banerjee D., Rai M. (2020). Social isolation in Covid-19: The impact of loneliness. Int. J. Soc. Psychiatry.

[8] Gerber M., Frankfurt S., Contractor A., Oudshoorn K., Dranger P., Brown L. (2018). Influence of Multiple Traumatic Event Types on Mental Health Outcomes: Does Count Matter?. J. Psychopathol. Behav. Assess..

[9] World Health Organization (2019). Mental Health in Emergencies.

[10] Zhu Y., Chen L., Ji H., Xi M., Fang Y., Li Y. (2020). The Risk and Prevention of Novel Coronavirus Pneumonia Infections Among Inpatients in Psychiatric Hospitals. Neurosci. Bull..

[11] Adhanom Ghebreyesus T. (2020). Addressing mental health needs: An integral part of COVID-19 response. World Psychiatry.

[12] Lee J.-Y., Kim S.-W., Kim J.-M. (2020). The Impact of Community Disaster Trauma: A Focus on Emerging Research of PTSD and Other Mental Health Outcomes. Chonnam Med. J..

[13] Cao W., Fang Z., Hou G., Han M., Xu X., Dong J., Zheng J. (2020). The psychological impact of the COVID-19 epidemic on college students in China. Psychiatry Res..

[14] Hawes M., Szenczy A., Klein D., Hajcak G., Nelson B. (2021). Increases in Depression and Anxiety Symptoms in Adolescents and Young Adults during the COVID-19 Pandemic. Psychol. Med..

[15] Hou F., Bi F., Jiao R., Luo D., Song K. (2020). Gender differences of depression and anxiety among social media users during the COVID-19 outbreak in China: A cross-sectional study. BMC Public Health.

[16] Antunes R., Frontini R., Amaro N., Salvador R., Matos R., Morouço P., Rebelo-Gonçalves R. (2020). Exploring lifestyle habits, physical activity, anxiety, and basic psychological needs in a sample of portuguese adults during covid-19. Int. J. Environ. Res. Public Health.

[17] Hyland P., Shevlin M., McBride O., Murphy J., Karatzias T., Bentall R., Martinez A., Vallières F. (2020). Anxiety and depression in the Republic of Ireland during the COVID-19 pandemic. Acta Psychiatr. Scand..

[18] Özdin S., Bayrak Özdin Ş. (2020). Levels and predictors of anxiety, depression, and health anxiety during COVID-19 pandemic in Turkish society: The importance of gender. Int. J. Soc. Psychiatry.

[19] Savitsky B., Findling Y., Ereli A., Hendel T. (2020). Anxiety and coping strategies among nursing students during the covid-19 pandemic. Nurse Educ. Pract..

[20] Ettman C., Abdalla S., Cohen G., Sampson L., Vivier P., Galea S. (2020). Prevalence of Depression Symptoms in US Adults Before and During the COVID-19 Pandemic. JAMA Netw. Open.

[21] Brown S.M., Doom J.R., Lechuga-Peña S., Watamura S.E., Koppels T. (2020). Stress and parenting during the global COVID-19 pandemic. Child Abuse Negl..

[22] Unützer J., Kimmel R.J., Snowden M. (2020). Psychiatry in the age of COVID-19. World Psychiatry.

[23] Hao F., Tan W., Jiang L., Zhang L., Zhao X., Zou Y., Hu Y., Luo X., Jiang X., McIntyre R. (2020). Do psychiatric patients experience more psychiatric symptoms during COVID-19 pandemic and lockdown? A case-control study with service and research implications for immunopsychiatry. Brain Behav. Immun..

[24] Tan B., Chew N., Lee G. (2020). Psychological Impact of the COVID-19 Pandemic on Health Care Workers in Singapore. Ann. Intern. Med..

[25] Sampaio F., Sequeira C., Teixeira L. (2020). Nurses’ Mental Health During the Covid-19 Outbreak: A Cross-Sectional Study. J. Occup. Med..

[26] Paulino M., Dumas-Diniz R., Brissos S., Brites R., Alho L., Simões M., Silva C. (2021). COVID-19 in Portugal: Exploring the immediate psychological impact on the general population. Psychol. Health Med..

[27] Gale T., Parker S. (2014). Navigating change: A typology of student transition in higher education. Stud. High. Educ..

[28] Frajerman A., Morvan Y., Krebs M., Gorwood P., Chaumette B. (2019). Burnout in medical students before residency: A systematic review and meta-analysis. Eur. Psychiatry.

[29] Hermetet C., Arnault É., Gaborit C., Coillot H., Florence A., Diot P., Colombat P., Rusch E., Grammatico-Guillon L. (2019). Prevalence and risk markers of anxiety and depression among health students. Presse Med..

[30] Akhtarul Islam M., Barna S.D., Raihan H., Nafiul Alam Khan M., Tanvir Hossain M. (2020). Depression and anxiety among university students during the COVID-19 pandemic in Bangladesh: A web-based cross-sectional survey. PLoS ONE.

[31] Sahu P. (2020). Closure of Universities Due to Coronavirus Disease 2019 (COVID-19): Impact on Education and Mental Health of Students and Academic Staff. Cureus.

[32] Glanz K., Rimer B.K., Viswanath K. (2015). Health Behavior: Theory, Research and Practice.

[33] Biggs A., Brough P., Drummond S. (2017). Lazarus and Folkman’s Psychological Stress and Coping Theory.

[34] Duggal D., Sacks-Zimmerman A., Liberta T. (2016). The Impact of Hope and Resilience on Multiple Factors in Neurosurgical Patients. Cureus.

[35] Folkman S. (2010). Stress, coping, and hope. Psychooncology.

[36] Ferreira M., Sofia R., Carreno D., Eisenbeck N., Jongenelen I., Cruz J. (2021). Dealing with the Pandemic of COVID-19 in Portugal: On the Important Role of Positivity, Experiential Avoidance, and Coping Strategies. Front. Psychol..

[37] Jarego M., Pimenta F., Pais-Ribeiro J., Costa R., Patrão I., Coelho L., Ferreira-Valente A. (2021). Do coping responses predict better/poorer mental health in Portuguese adults during Portugal’s national lockdown associated with the COVID-19?. Pers. Individ. Differ..

[38] Patel V., Flisher A., Hetrick S., McGorry P. (2007). Mental health of young people: A global public-health challenge. Lancet.

[39] Maia B., Dias P. (2020). Anxiety, depression, and stress in university students: The impact of COVID-19. Estud. De Psicol..

[40] Laranjeira C., Querido A., Marques G., Silva M., Simões D., Gonçalves L., Figueiredo R. (2021). COVID-19 pandemic and its psychological impact among healthy Portuguese and Spanish nursing students. Health Psychol. Res..

[41] Sun S., Goldberg S.B., Lin D., Qiao S., Operario D. (2021). Psychiatric symptoms, risk, and protective factors among university students in quarantine during the COVID-19 pandemic in China. Glob. Health.

[42] Raosoft Inc. (2004). RaoSoft® Sample Size Calculator. http://www.raosoft.com/samplesize.html.

[43] Apóstolo J.L., Mendes A.C., Azeredo Z.A. (2006). Adaptation to Portuguese of the Depression, Anxiety and Stress Scales (DASS). Rev. Lat. Am. Enfermagem..

[44] Lovibond P., Lovibond S. (1994). The structure of negative emotional states: Comparison of the Depression Anxiety Stress Scales (DASS) with the Beck Depression and Anxiety Inventories. Med. Biol. Eng. Comput..

[45] Du J., Mayer G., Hummel S., Oetjen N., Gronewold N., Zafar A., Schultz J. (2020). Mental Health Burden in Different Professions During the Final Stage of the COVID-19 Lockdown in China: Cross-sectional Survey Study. J. Med. Internet Res..

[46] Herth K. (1992). Abbreviated instrument to measure hope: Development and psychometric evaluation. J. Adv. Nurs..

[47] Viana A., Querido A., Dixe M., Barbosa A. (2010). Avaliação da esperança em cuidados paliativos: Tradução e adaptação transcultural do Herth Hope Index. Int. J. Dev. Educ. Psychol..

[48] Pais Ribeiro J., Morais R. (2010). Adaptação portuguesa da escala breve de coping resiliente. Psicol. Saúde Doenças.

[49] Sinclair V., Wallston K. (2004). The development and psychometric evaluation of the Brief Resilient Coping Scale. Assessment.

[50] World Medical Association (2013). World medical association declaration of Helsinki: Ethical principles for medical research involving human subjects. JAMA.

[51] Tabachnick B., Fidell L. (2013). Using Multivariate Statistics.

[52] Moreira P., Ferreira S., Couto B., Machado-Sousa M., Fernández M., Raposo-Lima C., Sousa N., Picó-Pérez M., Morgado P. (2021). Protective Elements of Mental Health Status during the COVID-19 Outbreak in the Portuguese Population. Int. J. Environ. Res. Public Health.

[53] Frontini R., Rebelo-Gonçalves R., Amaro N., Salvador R., Matos R., Mouroço P., Antunes R. (2021). The relationship between anxiety levels, sleep, and physical activity during COVID-19 lockdown: An exploratory study. Front. Psychol..

[54] Antunes R., Amaro N., Salvador R., Matos R., Morouço P., Rebelo-Gonçalves R., Frontini R. (2021). Higher physical activity levels may help buffer the negative psychological consequences of COVID-19 pandemic. Front. Psychol..

[55] Ramón-Arbués E., Gea-Caballero V., Granada-López J., Juárez-Vela R., Pellicer-García B., Antón-Solanas I. (2020). The Prevalence of Depression, Anxiety and Stress and Their Associated Factors in College Students. Int. J. Environ. Res. Public Health.

[56] Kebede M.A., Anbessie B., Ayano G. (2019). Prevalence and predictors of depression and anxiety among medical students in Addis Ababa, Ethiopia. Int. J. Ment. Health Syst..

[57] Tyrer P. (2020). COVID-19 health anxiety. World Psychiatry.

[58] Broche-Pérez Y., Fernández-Fleites Z., Fernández-Castillo E., Jiménez-Puig E., Vizcaíno-Escobar A., Ferrer-Lozano D., Martínez-Rodríguez L., Martín-González R. (2021). Anxiety, Health Self-Perception, and Worry About the Resurgence of COVID-19 Predict Fear Reactions Among Genders in the Cuban Population. Front. Glob. Womens Health.

[59] Evren C., Evren B., Dalbudak E., Topcu M., Kutlu N. (2020). Measuring anxiety related to COVID-19: A turkish validation study of the coronavirus anxiety scale. Death Stud..

[60] Bártolo A., Monteiro S., Pereira A. (2017). Estrutura fatorial e validade de construto da escala Generalized Anxiety Disorder 7-item (GAD-7) entre alunos universitários em Portugal. Cad. De Saude Publica.

[61] Wang C., Pan R., Wan X., Tan Y., Xu L., Ho C.S. (2020). Immediate Psychological Responses and 347 Associated Factors during the Initial Stage of the 2019 Coronavirus Disease (COVID-19) Epidemic among the 348 General Population in China. Int. J. Env. Res. Public Health.

[62] Horesh D., Kapel Lev-Ari R., Hasson-Ohayon I. (2020). Risk factors for psychological distress during the COVID-19 pandemic in Israel: Loneliness, age, gender and health status play an important role. Br. J. Health Psychol..

[63] Redlich Amirav D., Besor O., Amirav I. (2021). Hope During COVID-19 Lockdown. Cureus.

[64] Masten A.S., Wang M.C., Gordon E.W. (1994). Resilience in individual development: Successful adaptation despite risk and adversity. Educational Resilience in Innercity America: Challenges and Prospects.

[65] Molinero R., Zayas A., Ruíz-González P., Guil R. (2018). Optimism and resilience among university students. Int. J. Sch. Educ. Psychol..

[66] Alodhayani A., Almutairi K., Alshobaili F., Alotaibi A., Alkhaldi G., Vinluan J., Albedewi H., Al-Sayyari L. (2021). Predictors of Mental Health Status among Quarantined COVID-19 Patients in Saudi Arabia. Healthcare.

[67] Elmer T., Mepham K., Stadtfeld C. (2020). Students under lockdown: Comparisons of students’ social networks and mental health before and during the COVID-19 crisis in Switzerland. PLoS ONE.

[68] Lee J., Solomon M., Stead T., Kwon B., Ganti L. (2021). Impact of COVID-19 on the mental health of US college students. BMC Psychol..

[69] Oláh L.S., Kotowska I.E., Richter R., Doblhammer G., Gumà J. (2018). The New Roles of Men and Women and Implications for Families and Societies. A Demographic Perspective on Gender, Family and Health in Europe.

[70] AlFaris E., Irfan F., Qureshi R. (2016). Health professions students have an alarming prevalence of depressive symptoms: Exploration of the associated factors. BMC Med. Educ..

[71] Walsh F. (2020). Loss, and resilience in the time of COVID-19: Meaning making, hope, and transcendence. Fam. Process.

[72] Watts Isley J., Gonzales R., Drey J., Ritter E., Lawrence W., Rowe B., Sosa P. (2021). Adaptability, Change, Hope: Student Perspectives During the COVID-19 Pandemic. Am. J. Public Health.

[73] McDaid D. (2021). Viewpoint: Investing in strategies to support mental health recovery from the COVID-19 pandemic. Eur. Psychiatry.

[74] Yang C., Chen A., Chen Y. (2021). College students stress and health in the COVID-19 pandemic: The role of academic workload, separation from school, and fears of contagion. PLoS ONE.

[75] Stewart D.E., Appelbaum P.S. (2020). COVID-19 and psychiatrists’ responsibilities: A WPA position paper. World Psychiatry.

[76] Gao J., Zheng P., Jia Y. (2020). Mental health problems and social media exposure during COVID-19 outbreak. PLoS ONE.

[77] Sax L., Gilmartin S., Bryant A. (2003). Assessing response rate and nonresponse bias in web and paper surveys. Res. High. Ed..

[78] Rogelberg S., Stanton J. (2007). Understanding and Dealing with Organizational Survey Nonresponse. Organ. Res. Methods.

